# Emotional states as mediators between tinnitus loudness and tinnitus distress in daily life: Results from the “TrackYourTinnitus” application

**DOI:** 10.1038/srep20382

**Published:** 2016-02-08

**Authors:** Thomas Probst, Rüdiger Pryss, Berthold Langguth, Winfried Schlee

**Affiliations:** 1 Department of Psychology, University of Regensburg, Germany; 2 Department of Psychology and Psychotherapy, University of Witten/Herdecke, Germany; 3 Institute of Databases and Information System, Ulm University, Germany; 4 Department of Psychiatry and Psychotherapy, University of Regensburg, Germany

## Abstract

The psychological process how tinnitus loudness leads to tinnitus distress remains unclear. This cross-sectional study investigated the mediating role of the emotional state “stress level” and of the two components of the emotional state “arousal” and “valence” with N = 658 users of the “TrackYourTinnitus” smartphone application. Stress mediated the relationship between tinnitus loudness and tinnitus distress in a simple mediation model and even in a multiple mediation model when arousal and valence were held constant. Arousal mediated the loudness-distress relationship when holding valence constant, but not anymore when controlling for valence as well as for stress. Valence functioned as a mediator when controlling for arousal and even when holding arousal and stress constant. The direct effect of tinnitus loudness on tinnitus distress remained significant in all models. This study demonstrates that emotional states affect the process how tinnitus loudness leads to tinnitus distress. We thereby could show that the mediating influence of emotional valence is at least equally strong as the influence of stress. Implications of the findings for future research, assessment, and clinical management of tinnitus are discussed.

Chronic tinnitus, the perception of sound in the absence of any physical sound source for it, is highly prevalent. Between 4.4 and 15.1% suffer from prolonged tinnitus and 2.4% are plagued by tinnitus every day^1^. Furthermore, tinnitus patients are often impaired by psychological problems and disorders such as depression, anxiety, difficulties concentrating, and problems falling asleep^2^,^3^,^4^,^5^,^6^, particularly when they lack tinnitus acceptance^7^.

Comprehensive assessments of tinnitus involve the assessment of tinnitus loudness and of tinnitus-related distress. Tinnitus loudness can be assessed by rating scales or by psychophysiological measurements such as tinnitus loudness matching or minimal masking levels. As the reliability of tinnitus loudness-matching is relatively limited^8^, even when standardized procedures are used^9^, the assessment of tinnitus loudness by rating scales is more widespread. Further support for differentiating tinnitus loudness from tinnitus distress comes from neuroimaging studies, which demonstrate that these two components of tinnitus are encoded in different brain networks^10^,^11^,^12^,^13^. In line with these findings, clinical studies revealed different factors to be differently associated with tinnitus loudness and tinnitus distress. While loudness was correlated with permanent awareness and binaural localization, distress was related to depressivity, anxiety as well as somatic symptom severity^14^. Moreover, hyperacusis and resilience had a stronger impact on distress than on loudness^15^,^16^.

This fits with the clinical observation that tinnitus loudness and tinnitus distress are only moderately correlated (r = 0.45)^17^. In a survey with almost 5.000 respondents, most of the participants rated loudness and distress congruently, but 587 individuals experienced high loudness together with low distress and 28 subjects reported low loudness with high distress. Not feeling low/depressed and not considering oneself as victim of the noises were the psychological variables most strongly associated with low distress despite high loudness^17^. In a more recent study, it was investigated how the relationship between tinnitus loudness and tinnitus distress is mediated by tinnitus acceptance, depression, and anxiety^18^. The authors found that tinnitus acceptance and anxiety partially mediate the loudness-distress relationship and that the mediating effect is stronger for tinnitus acceptance than for anxiety. These two studies are to our knowledge the only two investigations on psychological and psychopathological processes that mediate the relationship between tinnitus loudness and tinnitus distress. A deeper understanding of the processes how the perception of loudness is related to distress is of utmost importance as it may guide the development of specific therapeutic interventions. In the area of pain research, for example, 12 studies investigated how psychological variables mediate the relationship between pain perception and pain-related problems^19^.

Andersson and Westin provided an overview on potential psychological mediators regarding tinnitus distress including stress levels^20^. Various studies have shown that stress plays a crucial role in tinnitus. For example, people reporting high tinnitus distress show elevated cortisol levels compared with people reporting low tinnitus distress as well as control persons^21^. Furthermore, stress has been shown to be particularly important regarding the transition from mild to severe tinnitus^22^. Moreover, tinnitus patients report more strain than healthy control persons in stress tests^23^. Conceptually, stress can be described as an emotional state that is considered to be composed of an arousal and a valence component^24^. The component arousal describes the emotional state on a continuum between calm and excitement, while the component valence describes the emotional state on a continuum varying from positive to negative feelings. These components are usually measured by the “Self-Assessment Manikin” (SAM)^25^. As neither the emotional state stress level nor the two components of the emotional state (arousal/valence) were evaluated as mediators between tinnitus loudness and tinnitus distress, we investigated three research questions: 1) Is the loudness-distress relationship mediated by the emotional state stress level? 2) Is the loudness-distress relationship mediated by the arousal and/or the valence component of the emotional state and - if both components are mediators - is the mediating effect stronger for one component?

In general, a stressful event can, like all emotional states, be described on the dimensions of arousal and valence. Therefore, the question arises if stress adds an additional component to the mediation model and the third research question was designed with an explorative character to examine, whether other variables beyond arousal and valence should be considered as relevant components of the emotional state stress level in future research. 3) Is the emotional state stress level still a mediator when the two components of the emotional state - arousal and valence - are held constant?

## Method

### Participants

Participants were N = 658 users (test-users were excluded), who completed the questionnaire of the “TrackYourTinnitus” application at least once between 11/2013 and 8/2014. The sample description is summarized in Table 1. Missing values amounted to 1% for each variable.

### Material

The “TrackYourTinnitus” (www.trackyourtinnitus.org) platform consists of a website for registration, two mobile applications (for iOS and Android), and a SQL database for the central storage of the collected data^26^. The smartphone applications allow to track the individual tinnitus perception by self-assessment questionnaires about the tinnitus loudness, tinnitus distress, emotional valence and arousal, stress level and level of concentration. Ecological momentary assessment by handheld devices has been recently introduced as an innovative methodological approach for the study of chronic health problems characterized by high temporal and/or situational variability in symptoms and distress^26^,^27^,^28^. The users were asked to rate all these variables for the current moment (e.g. “How stressful is your tinnitus *right now*”). Visual analog scales were used to measure tinnitus loudness, tinnitus distress, stress and concentration, while the SAM^25^ was used for the measurement of emotional arousal and valence. The patients were asked to complete the app questionnaires at different time points during the day to allow a tinnitus assessment during their daily routine. In the standard setting, the notifications for filling out the questionnaires were scheduled on a random basis. While filling out the questionnaire, the background sound level was measured using the built-in microphone of the smartphone. The “TrackYourTinnitus” platform went online in April 2014 and is available in English and German. The data set used for the current analysis was exported in August 2015.

The material and the methods were approved by the Ethics Committee of the University Clinic of Regensburg and were carried out in accordance with the approved guidelines. The users’ of the app were informed that the data will be used for scientific analyses (informed consent).

### Statistical analysis

**Figure 1:**
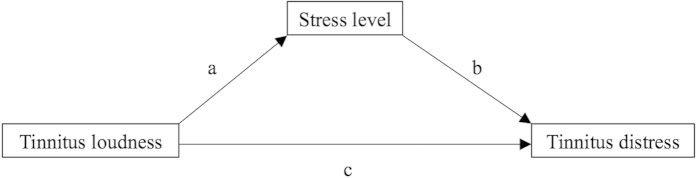
Path diagram of the simple mediation model: Investigating stress level as a mediator between tinnitus loudness and tinnitus distress. c = direct effect of tinnitus loudness on tinnitus distress; ab = indirect effect of tinnitus loudness on tinnitus distress through stress level.

SPSS 23 was used to perform the statistical analyses. As descriptive statistics, means (M), standard deviations (SD), percentages (%), and frequencies (n) were computed. To investigate how emotional states mediate the relationship between tinnitus loudness and tinnitus distress, the PROCESS^29^ macro for SPSS was used. Only the data from the first assessment point (first use of the app) of the participants was analyzed, because PROCESS was not designed for repeated assessments. Therefore, all assessments except the first assessment point of a user were deactivated for the mediation analyses. Within the macro, model 4 and 10.000 bias corrected bootstrap samples were selected. A 95% confidence level was chosen to apply a p-value of 0.05. Using these settings, three analyses were performed. In all three mediation analyses, tinnitus loudness was selected as independent variable and tinnitus distress was selected as outcome variable. To address our first research question, stress level was added as the mediator variable in a *simple mediation model*. For the second research question, arousal and valence were selected as mediator variables in a *multiple mediator model*. Furthermore, the option to compare indirect effects was activated in the multiple mediator model to compare the mediating effects of arousal and valence. To examine the third research question, arousal, valence, and stress level were selected as mediator variables in another *multiple mediator model*. Again, the option to compare indirect effects was activated. Figures 1 display the path diagrams of the mediation analyses.

## Results

### Results for the simple mediation analysis – Investigating stress level as a mediator between tinnitus loudness and tinnitus distress

N = 604 users provided data on current tinnitus loudness, current tinnitus distress, as well as on the current stress level when they used the “TrackYourTinnitus” application for the first time (first assessment point). Hence, n = 54 users (8.2% of N = 658) had to be excluded due to missing data.

Table 2 provides the results of the analysis done with PROCESS. It can be seen that the direct effect of tinnitus loudness on tinnitus distress was significantly different from zero (p < 0.05). Therefore, tinnitus loudness had an impact on tinnitus distress even when controlling for the current stress level. The 95%-confidence interval of the bootstrap results revealed that the indirect effect of tinnitus loudness on tinnitus distress through the current stress level was different from zero (lower level: 0.066; upper level: 0.135) indicating that the stress level partially mediated the relationship between tinnitus loudness and tinnitus distress.

### Results for the first multiple mediation analysis – Investigating arousal and valence as mediators between tinnitus loudness and tinnitus distress

N = 606 users completed the questions on current tinnitus loudness, current tinnitus distress, current arousal, and current valence at their first app use. Thus, n = 52 (7.9% of N = 658) were excluded from this mediation analysis due to missing data. Table 3 displays the results of the PROCESS output. As can be seen, the direct effect of tinnitus loudness on tinnitus distress was significantly different from zero (p < 0.05). This indicates that tinnitus loudness affected tinnitus distress even when holding the current arousal and current valence constant. However, the 95%-confidence intervals of the bootstrap results indicate that the indirect effects were different from zero for arousal (lower level: 0.003; upper level: 0.027) as well as for valence (lower level: 0.068; upper level: 0.135). Therefore, arousal and valence both partially mediated the relationship between tinnitus loudness and tinnitus distress. The comparison of both indirect effects using a 95%-confidence interval revealed that the indirect effect of valence was stronger than the indirect effect of arousal (lower level: –0.126; upper level: –0.054).

### Results for the second multiple mediation analysis – Investigating stress level, arousal, and valence as mediators between tinnitus loudness and tinnitus distress

At the first app use, only n = 592 users provided data on the items evaluating current tinnitus loudness, current tinnitus distress, current stress level, current arousal, and current valence. Hence, n = 66 (10.0% of N = 658) had to be excluded from this mediation analysis due to missing data. The PROCESS output is summarized in Table 4. Again, the direct effect of tinnitus loudness on tinnitus distress was significantly different from zero (p < 0.05) meaning that tinnitus loudness predicted tinnitus distress even when controlling for all the emotional states investigated in this study (stress level, arousal, and valence). The 95%-confidence intervals of the bootstrap results showed that the indirect effects were different from zero for stress level (lower level: 0.030 upper level: 0.089) as well as for valence (lower level: 0.054; upper level: 0.118), but not for arousal (lower level: –0.005; upper level: 0.016). The mediating effect of stress level was neither higher nor lower than the mediating effect of valence (lower level: –0.073; upper level: 0.018).

## Discussion

This cross-sectional study examined the role of emotional states on the relationship between tinnitus loudness and tinnitus distress. Specifically, we investigated whether the emotional state stress level is a mediator of the loudness-distress relationship and which component of the emotional state – arousal or valence – is a stronger mediator. In an explorative analysis, we examined whether the emotional state stress level is still a significant mediator when the two components of the emotional state - arousal and valence - are held constant. The aim of this analysis was to explore whether more aspects than arousal and valence should be considered as components of the emotional state stress level in future research.

As expected, the results of the first mediation analysis revealed that the stress level significantly mediates the association between tinnitus loudness and tinnitus distress. However, loudness significantly predicted distress even when the mediator stress level was held constant. Therefore, the individually reported state of stress level is a partial mediator between tinnitus loudness and distress. More specifically, the positive estimate of 0.328 for path a (Fig. 1) implicates that increases of tinnitus loudness result in an increase of the individual stress level. Furthermore, a general increase of stress leads to increases in tinnitus distress (path b estimate of 0.297). Based on these data one would expect that a clinical intervention that reduces the stress level of the tinnitus patient would also lead to a reduction of tinnitus distress. However, it has to be mentioned here that the direct effect of tinnitus loudness on tinnitus distress (path c estimate 0.652) is much stronger indicating limitations for therapeutic interventions that reduce only the general stress level without changing the tinnitus loudness.

**Figure 2:**
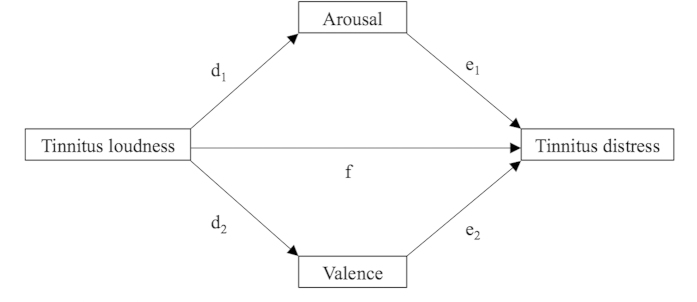
Path diagram of the first multiple mediation model: Investigating arousal and valence as mediators between tinnitus loudness and tinnitus distress. f = direct effect of tinnitus loudness on tinnitus distress; d_1_e_1_ = indirect effect of tinnitus loudness on tinnitus distress through arousal; d_2_e_2_ = indirect effect of tinnitus loudness on tinnitus distress through valence.

Furthermore, we investigated the mediating effects of the two components of the emotional state – arousal and valence – as mediators between loudness and distress in our second mediation analysis. Again, the loudness-distress association remained significant when holding the mediators, arousal and valence, constant. Nevertheless, arousal and valence were both significant mediators and thus functioned as partial mediators. When contrasting the two mediators regarding their impact on the loudness-distress relationship, the effect of valence was significantly stronger than the effect of arousal. In detail, we observed a positive influence of tinnitus loudness on emotional arousal (d_1_ path estimate 0.128, Fig. 2) meaning that the louder the tinnitus the stronger the emotional arousal. In addition, stronger arousal leads to higher tinnitus-related distress (e_1_ path, estimate 0.093). This is in line with a recent publication reporting that a blockade of the superior cervical sympathetic ganglion leads to a reduction of tinnitus symptoms^30^. Thus, interventions leading to a reduction of arousal should – to a certain extend – reduce tinnitus distress. The emotional component valence, however, was inversely associated with tinnitus. Higher values of the valence component represent positive emotional states, while smaller values indicated negative emotions. Thus, the negative estimate between tinnitus loudness and valence (d_2_ path, estimate –0.258) implies that higher tinnitus loudness is linked to emotions that are more negative. Interestingly, the influence of loudness on valence was apparently twice as strong as the effect of loudness on arousal (d_1_ path estimate 0.128). Moreover, the general rating of emotional valence was negatively associated with tinnitus distress (e_2_ path estimate of –0.381), indicating lower tinnitus distress in moments of positive emotional states. We want to stress at this point that the influence of valence on tinnitus distress was apparently four times stronger than the impact of arousal on tinnitus distress (e_1_ path estimate 0.093 vs. e_2_ path estimate –0.381). This highlights the importance of the emotional components in the clinical treatment of tinnitus patients. Concerning the higher relevance of the valence component, it has been found that valence is more strongly related to the activity of the amygdala than arousal^31^. As imaging studies have identified the amygdala as a key region in tinnitus pathophysiology^32^,^33^, it could be speculated that the amygdala plays a role in the process how loudness leads to distress. Recently, it has been attempted in a large EEG study to identify the neuronal correlates of tinnitus loudness, tinnitus distress and the connection between these components^12^. By analyzing data of 317 tinnitus patients and 256 healthy subjects with independent component analysis, two major brain networks could be identified: one reflecting tinnitus loudness, the other tinnitus distress. One specific connection between the two networks (from the subgenual anterior cingulate cortex to the parahippocampus) was detectable only in those patients that were distressed by their tinnitus and was therefore interpreted as the link between tinnitus loudness and distress. Similarly, one could try to pinpoint neuronal correlates for valence and arousal. Although arousal and valence mediated the relationship between tinnitus loudness and tinnitus distress, tinnitus loudness still predicted tinnitus distress when the mediators were held constant. Hence, clinical interventions targeting arousal and valence might reduce tinnitus distress to a certain extent, but loudness would still evoke distress.

The third research question of this paper was explorative and contrasted the emotional state stress level as well as both components of the emotional state - arousal and valence - in one multiple mediator model. The result that the emotional state stress level still was a mediator even when controlling for both components of the emotional state - arousal and valence - implies that more facets than arousal and valence contributed to the effect of the emotional state stress level. One relevant other facet of the stress level might for example be the current feeling of dominance (i.e. sense of control), which is integrated in the SAM^25^ but not in the “TrackYourTinnitus” app. Moreover, the two processes - cognitive appraisal and coping - highlighted in the transactional stress model^34^ might be of importance in this context. Interestingly, this explorative analysis also revealed that valence still mediated the relationship between tinnitus loudness and tinnitus distress even when controlling for stress level and arousal. This indicates that beyond stress level and arousal other components contribute to the effect of valence (e.g. satisfaction of needs). Adding more items to the “TrackYourTinnitus” app (e.g. dominance, cognitive appraisal, coping) would allow to investigate the additional value of these variables in future studies. However, it should be kept in mind that adding too much items could entail negative effects regarding the use of the app in the daily routine.

Furthermore, the third analysis allows a comparison between the mediating effect of stress and emotional valence. The influence for the stress pathway was estimated with 0.056 (95%-confidence interval from 0.030 to 0.089), while the influence for the emotional valence was estimated stronger with 0.083 (95%-confidence interval from 0.054 to 0.118), however, slightly not significant. This analysis shows that the influence of the emotional valence is at least equally - if not more - important as the stress level for understanding the fluctuations of the individual tinnitus distress due to tinnitus loudness.

Our result that tinnitus loudness predicts tinnitus distress even when controlling for psychological state variables is in line with the results of a study showing that tinnitus loudness significantly affected tinnitus distress when controlling for the psychological non-state variables acceptance, depression, and anxiety^18^. Taken together, loudness seems to lead to distress independently of the studied psychological state and non-state variables. Nevertheless, future research should try to identify further psychological variables that mediate the relationship between tinnitus loudness and tinnitus distress. Further longitudinal studies should also investigate whether the use of specific psychotherapeutic interventions for modifying stress, arousal and valence has an impact on tinnitus distress and on the relationship between tinnitus loudness and distress.

As our results rely solely on self-ratings, the findings have to be interpreted with caution. To overcome this limitation, future studies could use psychoacoustic measurements for complementary assessment of tinnitus loudness and also quantify stress level, arousal, and valence by measuring physiological correlates such as skin conductance, cortisol level, or heart rate^35^. A further limitation is that we only investigated the emotional state stress level, and both components of the emotional state – arousal and valence – as mediators of the loudness-distress relationship, while several other aspects have been found to be also relevant for tinnitus distress: e. g., catastrophizing, negative cognitions, acceptance, anxiety, depression, age, gender, duration of tinnitus, age at tinnitus onset, level of education, and somatic complaints^18^,^36^,^37^,^38^,^39^,^40^,^41^.

Despite these limitations, we provided first empirical evidence that the current stress level and both components of the emotional state – with valence surpassing arousal – partially mediate the relationship between tinnitus loudness and tinnitus distress. The results underline the importance of reducing the patient’s stress level and improving arousal and valence in the clinical routine, but also show the limits of these interventions. Moreover, the study demonstrates the feasibility to investigate psychological aspects of tinnitus by using a smart-phone app. This method has the advantages of a higher ecological validity as assessments take place in a natural environment and not at a hospital or a research institute^26^,^27^,^28^. Furthermore, the sample is not restricted to patients seeking help and therefore presumably more representative of the total population of people with tinnitus than a clinical cohort.

## Additional Information

**How to cite this article**: Probst, T. *et al.* Emotional states as mediators between tinnitus loudness and tinnitus distress in daily life: Results from the “TrackYourTinnitus” application. *Sci. Rep.* **6**, 20382; doi: 10.1038/srep20382 (2016).

## Figures and Tables

**Figure 3:**
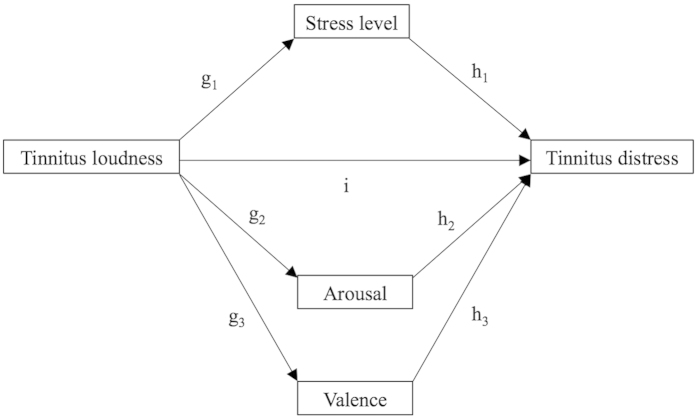
Path diagram of the second multiple mediation model: Investigating stress level, arousal, and valence as mediators between tinnitus loudness and tinnitus distress. i = direct effect of tinnitus loudness on tinnitus distress; g_1_h_1_ = indirect effect of tinnitus loudness on tinnitus distress through stress; g_2_h_2_ = indirect effect of tinnitus loudness on tinnitus distress through arousal; g_3_h_3_ = indirect effect of tinnitus loudness on tinnitus distress through valence.

**Table 1:** Sample description.

**Variable**		Descriptivestatistics
Gender: n (%)	female	179 (27.5)
	male	472 (72.5)
Age in years: M (SD)		43.02 (14.19)
Time since onset in years: M (SD)		8.87 (11.48)
Family history: n (%)	yes	150 (23.1)
	no	500 (76.9)
Variability: n (%)	yes	491 (75.7)
	no	158 (24.3)
Relation to onset: n (%)	change in hearing	72 (11.0)
	stress	188 (28.8)
	loud blast of sound	106 (16.2)
	head trauma	26 (4.0)
	whiplash	16 (2.5)
	other	245 (37.5)

**Table 2:** Results of the simple mediation analysis investigating stress level as a mediator between tinnitus distress and tinnitus loudness.

**Normal theory test**				
	**Coefficient**	**SE**	**t**	**p**
Effect of tinnitus loudness on stress level (a path)	0.328	0.038	8.657	0.000
Effect of stress level on tinnitus distress (b path)	0.297	0.031	9.547	0.000
Direct effect of tinnitus loudness on tinnitus distress (c path)	0.652	0.031	21.26	0.000
**Bootstrap results for indirect effects**				
	Bootstrap estimate		95% confidence interval	
	Estimate	SE	Lower	Upper
Indirect effect of tinnitus loudness on tinnitus distressthrough stress level (a × b path)	0.098	0.018	0.066	0.135

**Table 3:** Results of the first multiple mediation analysis investigating arousal and valence as mediators between tinnitus distress and tinnitus loudness.

**Normal theory test**				
	**Coefficient**	**SE**	**t**	**p**
Effect of tinnitus loudness on arousal (d_1_ path)	0.128	0.037	3.432	0.001
Effect of arousal on tinnitus distress (e_1_ path)	0.093	0.032	2.897	0.004
Effect of tinnitus loudness on valence (d_2_ path)	−0.258	0.029	−8.839	0.000
Effect of valence on tinnitus distress (e_2_ path)	−0.381	0.041	−9.353	0.000
Direct effect of tinnitus loudness on tinnitus distress (f path)	0.635	0.030	21.067	0.000
**Bootstrap results for indirect effects**				
	Bootstrap estimate		95% confidence interval	
	Estimate	SE	Lower	Upper
Total indirect effect of tinnitus loudness on tinnitus distress	0.110	0.018	0.078	0.148
Indirect effect of tinnitus loudness on tinnitus distress through arousal (d_1_ × e_1_ path)	0.012	0.006	0.003	0.027
Indirect effect of tinnitus loudness on tinnitus distress through valence (d_2_ × e_2_ path)	0.098	0.017	0.068	0.135
Specific indirect effect contrast (arousal – valence)	−0.087	0.018	−0.126	−0.054

**Table 4:** Results of the second multiple mediation analysis investigating stress level, arousal, and valence as mediators between tinnitus distress and tinnitus loudness.

**Normal theory test**				
	**Coefficient**	**SE**	**t**	**p**
Effect of tinnitus loudness on stress level (g_1_ path)	0.312	0.038	8.246	0.000
Effect of stress level on tinnitus distress (h_1_ path)	0.179	0.037	4.852	0.000
Effect of tinnitus loudness on arousal (g_2_ path)	0.137	0.038	3.631	0.000
Effect of arousal on tinnitus distress (h_2_ path)	0.028	0.035	0.793	0.428
Effect of tinnitus loudness on valence (g_3_ path)	−0.267	0.030	−9.028	0.000
Effect of valence on tinnitus distress (h_3_ path)	−0.310	0.043	−.175	0.000
Direct effect of tinnitus loudness on tinnitus distress (i path)	0.601	0.031	19.668	0.000
**Bootstrap results for indirect effects**				
	Bootstrap estimate		95% confidence interval	
	Estimate	SE	Lower	Upper
Total indirect effect of tinnitus loudness on tinnitus distress	0.143	0.020	0.105	0.185
Indirect effect of tinnitus loudness on tinnitus distress through stress level (g_1_ × h_1_ path)	0.056	0.015	0.030	0.089
Indirect effect of tinnitus loudness on tinnitus distress through arousal (g_2_ × h_2_ path)	0.004	0.005	−0.005	0.016
Indirect effect of tinnitus loudness on tinnitus distress through valence (g_3_ × h_3_ path)	0.083	0.016	0.054	0.118
Specific indirect effect contrast 1 (stress - arousal)	0.052	0.017	0.023	0.090
Specific indirect effect contrast 2 (stress - valence)	−0.027	0.023	−0.073	0.018
Specific indirect effect contrast 3 (arousal- valence)	−0.079	0.017	−0.116	−0.048
